# Mental health of children with epilepsy compared to their peers: population-based cohort from linked primary and secondary healthcare record in England

**DOI:** 10.23889/ijpds.v9i5.2845

**Published:** 2024-09-18

**Authors:** Millie Wagstaff, Ania Zylbersztejn, Sophie Bennett

**Affiliations:** 1 UCL Great Ormond Street Institute of Child Health; 2 NIHR Great Ormond Street Hospital Biomedical Research Centre; 3 KCL Institute of Psychiatry, Psychology and Neuroscience

## Background

Children with epilepsy have higher rates of internalising mental health (MH) conditions compared to their peers, although available evidence is limited by small and selective samples. We used linked primary care and hospital admission records covering approximately 4.42% of English population to describe incidence of mental health problems in children with epilepsy compared to their peers.

## Methods

We developed a cohort of young people aged 7-17 years old between 2010-2019, with minimum 1 year of follow-up until death, 18th birthday or 2020 using linked data from the Clinical Practice Research Datalink and Hospital Episodes Statistics. Epilepsy (exposure), depression and anxiety (outcomes) were identified using a combination of diagnostic codes, and primary care prescribing records. We estimated incident rate ratios (IRR) for anxiety/depression/any MH condition for children with epilepsy compared to their peers using negative binomial regression adjusted for sex, age, and area-level deprivation.

## Results

Study cohort included 510,177 children and young people, of whom 3,011 (0.59%) had epilepsy. Children with epilepsy had higher incidence of any MH condition (IRR: 1.39, 95% CI: 1.11–1.73) and anxiety (IRR: 1.38, 95% CI: 1.06–1.77), with differences in depression being non-significant (IRR: 1.25, 95% CI: 0.92–1.66).

## Discussion

Children with epilepsy have higher rates of MH problems than their peers. Further work will examine if this can be partially explained by increased opportunity for MH diagnosis due to more healthcare contacts. Our findings emphasise the importance of integrated care models that address both neurological and psychological aspects of epilepsy management.

